# The Metabolite Urolithin-A Ameliorates Oxidative Stress in Neuro-2a Cells, Becoming a Potential Neuroprotective Agent

**DOI:** 10.3390/antiox9020177

**Published:** 2020-02-21

**Authors:** Guillermo Cásedas, Francisco Les, Carmen Choya-Foces, Martín Hugo, Víctor López

**Affiliations:** 1 Facultad de Ciencias de la Salud, Universidad San Jorge, 50830 Villanueva de Gállego (Zaragoza), Spain; gcasedas@usj.es (G.C.); fles@usj.es (F.L.); 2 Instituto Agroalimentario de Aragón-IA2 (CITA-Universidad de Zaragoza), 50059 Zaragoza, Spain; 3 Unidad de Investigación, Hospital Universitario Santa Cristina, Instituto de Investigación Sanitaria Princesa (IIS-IP), E-28009 Madrid, Spain; carmenchoyaf@gmail.com (C.C.-F.); martin.hugo@salud.madrid.org (M.H.)

**Keywords:** urolithin A, pomegranate, ellagic acid, dietary polyphenols, neuroprotection, peroxiredoxins

## Abstract

Urolithin A is a metabolite generated from ellagic acid and ellagitannins by the intestinal microbiota after consumption of fruits such as pomegranates or strawberries. The objective of this study was to determine the cytoprotective capacity of this polyphenol in Neuro-2a cells subjected to oxidative stress, as well as its direct radical scavenging activity and properties as an inhibitor of oxidases. Cells treated with this compound and H_2_O_2_ showed a greater response to oxidative stress than cells only treated with H_2_O_2_, as mitochondrial activity (MTT assay), redox state (ROS formation, lipid peroxidation), and the activity of antioxidant enzymes (CAT: catalase, SOD: superoxide dismutase, GR: glutathione reductase, GPx: glutathione peroxidase) were significantly ameliorated; additionally, urolithin A enhanced the expression of cytoprotective peroxiredoxins 1 and 3. Urolithin A also acted as a direct radical scavenger, showing values of 13.2 μM Trolox Equivalents for Oxygen Radical Absorbance Capacity (ORAC) and 5.01 µM and 152.66 µM IC_50_ values for superoxide and 2,2-diphenyss1-picrylhydrazyl (DPPH) radicals, respectively. Finally, inhibition of oxidizing enzymes, such as monoamine oxidase A and tyrosinase, was also detected in a dose-dependent manner. The cytoprotective effects of urolithin A could be attributed to the improvement of the cellular antioxidant battery, but also to its role as a direct radical scavenger and enzyme inhibitor of oxidases.

## 1. Introduction

Urolithins are natural polyphenolic compounds obtained from ellagic acids and ellagitannins by the gut microbiota. Ellagic acid is a phenolic antioxidant compound present in numerous fruits, such as pomegranates, strawberries, or nuts. Ellagitannins are a class of polymeric ellagic acid derivatives also present in pomegranates (punicalagins and punicalins) and other sources, such as oak or chestnut wood (castalagin, castalin, roburin A, grandinin) or *Melaleuca quinquenervia* leaves (castalin and grandinin) [1,2,3].

The beneficial properties of these plants and foods seem to be in relation with these polyphenolic metabolites; however, the metabolism of polyphenols from food seems to be insufficient to achieve adequate levels of urolithins in the body. In addition, it has been proven that apparently beneficial foods such as pomegranates have had a lot of interindividual variability due to the different urolithin metabotypes present in the population [4]. In fact, only 1 in 3 people have the right microbiota to perform this metabolism with maximum efficiency [5].

Therefore, it is very important to evaluate the activity of isolated urolithins as potential therapeutic agents. In addition, the use if urolithin A in humans and the safety profile of this compound have been widely evaluated, with no adverse effects on health observed [6]. 

Although urolithins are a group of metabolites, urolithin A (UA), also known as 3,8-dihydroxyurolithin, is one of the most representative compounds. There are numerous studies that demonstrate an important role of this compound in metabolic syndrome, improving cardiovascular function, decreasing the formation of triglycerides, inhibiting enzymes such as lipase or glucosidase, or relieving insulin resistance [7,8,9]. It has also been observed that UA may have an important role in the prevention of certain cancers, such as colorectal or prostate cancers [10,11]. UA also has an important role at the mitochondrial level, being able to activate mitophagy and prolonging lifespan in *Caenorhabditis elegans* worms, as well as beneficial mitochondrial effects in the skeletal muscle [12,13].

The set of all these beneficial properties for health may be due to the antioxidant capacity of polyphenols. However, there are few studies that link the antioxidant properties of this metabolite with a potential therapeutic activity in neurodegenerative diseases, where the redox status is essential. Therefore, the objective of this study was to evaluate whether urolithin A has antioxidant and neuroprotective effects using Neuro-2a cells and other in vitro models involving the use of central nervous system (CNS) enzymes or free radicals.

## 2. Materials and Methods 

### 2.1. Reagents and Chemicals

**Figure 1:**
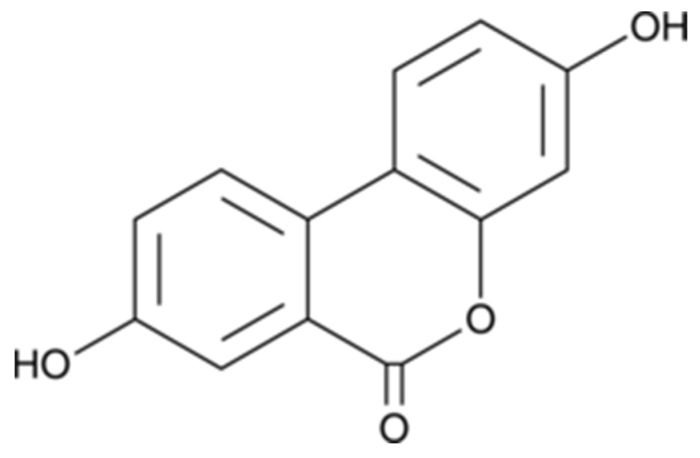
Structure of Urolithin A. Synonyms: 3,8-Dihydroxyurolithin; 3,8-Hydroxydibenzo-α-pyrone; 2′,7-Dihydroxy-3,4-benzocoumarin; δ-Lactone 2′,4,4′-Trihydroxy-2-biphenylcarboxylic acid.

Urolithin A (3,8-dihydroxyurolithin) (Figure 1) was purchased from Toronto Research Chemicals (TRC, Toronto, ON, Canada). Neuro-2a (N2a) cell line was provided from the American Type Culture Collection (ATCC, Manassas, VA, USA), while Monoamine oxidase A (MAO-A), 5,5′-dithiobis-(2-nitrobenzoic acid) (DTNB), Tris, galantamine, levodopa (l-DOPA), tyramine, horseradish peroxidase, 2,2′-azobis(2-methyl-propionamidine)-di-hydrochloride (AAPH), hydrogen peroxide (30% *w*/*w*), 2,4,6-Tris(2-pyridyl)-1,3,5-triazine (TPTZ), 2′,7′-dichloro-dihy-drofluorescein diacetate (DCFH-DA), 3-(4,5-Dimethyl-2-thiazolyl)-2,5- diphenyltetrazolium bromide (MTT), 6-hydroxy-2,5,7,8-tetra-methylchromane-2-carboxylic acid (Trolox), bovine serum albumin (BSA), vanillic acid, 4-aminoantipyrine, penicillin, streptomycin, tyrosinase, thiobarbituric acid (TBA), trichloroacetic acid (TCA), bicinchoninic acid (BCA), and pyrogallol were obtained from Sigma-Aldrich (Madrid, Spain). Clorgyline and α-kojic were from Cymit química (Barcelona, Spain). Na_2_CO_3_, HCl, NaCl, dimethyl sulfoxide (DMSO), MeOH, and potassium phosphate were supplied from Panreac (Barcelona, Spain). Dulbecco’s modified Eagle’s medium (DMEM), phosphate-buffered saline (PBS), and fetal bovine serum (FBS) were acquired from Gibco (Invitrogen, Paisley, UK).

### 2.2. Cytoprotective Properties of Urolithin A in Neuro-2a Cells

#### 2.2.1. Neuro-2a Cell Culture and Treatments with Urolithin A and Hydrogen Peroxide

Neuro-2a cells (ATCC^®^ CCL-131^™^) were thawed and cultured in 10% FBS-supplemented DMEM and 1% penicillin-streptomycin, seeded in a T175 flask, and placed into the incubator (5% CO_2_, 37 °C). Once they reached the state of confluence, they were sub-cultured in a 96-well plate at a density of 1 × 10^4^ cells/well in DMEM (10%) and incubated (37 °C, 5% CO_2_) for 24 h before the treatment.

Stock solutions of urolithin A (438 mM) were prepared in sterilized PBS containing 1% DMSO (final concentration in the cells less than 0.1%). The sample was vortexed and filtered with a 0.22-μm syringe filter. Five dilution series were done from the stock solution. Stock solutions of hydrogen peroxide (1000 μM) were prepared and incorporated into the cells at a final concentration of 250 μM (45 min) to induce oxidative stress in the cells.

#### 2.2.2. Mitochondrial Activity in Neuro-2a Cells Subjected to Oxidative Stress after Urolithin A Treatment

Cells were cultured in 96-well plates at a concentration of 1 × 10^4^ cells per well. The cytotoxicity of urolithin A (0.5 μM–20 μM) was measured after 24 h by adding MTT (3-(4,5-dimethylthiazol-2-yl)-2,5-diphenyltetrazolium bromide) to the cell culture. Additionally, this assay can be performed to assess the potential cytoprotective effect of urolithin A after inducing a neuronal injury with 250 μM hydrogen peroxide for 45 min. After treatments (24 h exposition to urolithin, 45 min to hydrogen peroxide), cells were incubated for an additional 24 h; DMEM was then removed from every well and replaced with MTT solution (2 mg/mL in DMEM) and incubated at 37 °C for 3 h. Finally, MTT solution was removed and 100 μL of DMSO was added in each well to dissolve formazan crystals. Via a Synergy H1 Hybrid Multi-Mode Reader (Biotek, Bad Friedrichshall, Germany), the absorbance was read at 550 nm. Experiments were carried out in different weeks and diverse passages and expressed as percentage of control. 

#### 2.2.3. ROS Production in Neuro-2a Cells Subjected to Oxidative Stress after Exposition to Urolithin A

Neuro-2a cells were seeded in a 96-well plate. After 24 h, the medium was replaced with PBS supplemented with glucose and DCFH-DA (2,7-di-chloro-dihydrofluorescein diacetate, 0.01 M) for 30 min at 37 °C. After this time, PBS was removed and cells were washed twice with new PBS and treated with different concentrations of urolithin A (0.5–4 μM), as well as hydrogen peroxide (250 μM). The absorbance was checked at 480 nm (λ_excitation_) and 520 nm (λ_emission_) wavelengths [14]. The kinetic was performed over 90 min in a Synergy H1 Hybrid Multi-Mode Reader (Winooski, VT, USA). Results were represented as a percentage of intracellular ROS production (100% of control).

#### 2.2.4. Lipid Peroxidation in Neuro-2a Cells Subjected to Oxidative Stress after Exposition to Urolithin A (TBARS assay)

Lipid peroxidation was assessed using the protocol by Mitsuru Uchiyama [15]. First, 100 μL of thawed pellets were placed into 200 μL of TBA–TCA–HCl cocktail and subsequently boiled at 100 °C for 10 min to accelerate the reaction. The reaction was broken by placing the samples on ice and stunning them in a vortex three times for 20 min and centrifuged at 4 °C (3000 rpm, 10 min). The obtained supernatant was placed in three different wells of a 96-well plate and absorbance was read at 530 nm using a Synergy H1 Hybrid Multi-Mode Reader.

#### 2.2.5. Activity of Antioxidant Enzymes in Neuro-2a Cells Subjected to Oxidative Stress after Exposition to Urolithin A

Before analyzing the activity of antioxidant enzymes, the protein content of Neuro-2a cells was calculated by colorimetric bicinchoninic acid (BCA) assay and normalized lysis buffer for 20 min (Ethylenediaminetetraacetic acid (EDTA) 1 mM, Tris 25 mM, NaCl 150 mM and 0.1% Triton; pH = 7.4). Moreover, leupeptin, pepstatin, and phenylmethylsulfonyl fluoride (PMSF) proteinase inhibitors (20, 10, and 35 μL/mL, respectively) were aggregated into the buffer. Finally, supernatant was kept for the experiments and the precipitate was discard.

The activity of catalase was measured as follows: hydrogen peroxide (1970 μL, 15 mM) was prepared in sodium phosphate buffer (pH = 7.5) supplemented with 30 μL of supernatant [16]; the mixture was prepared in a quartz cuvette, measuring the absorbance for 30 secs at 240 nm, using a Shimadzu Spectrophotometer UV-1800 (Duisburg, Germany). The activity of the enzyme was expressed following the next equation: Catalase activity = ((∆Abs/min) × 2 × F)/(0.0436 × Vs × C)

43.6 mL nmol^−1^ cm^−1^: molar extinction coefficient of H_2_O_2_

F: dilution factor

C: Protein concentration (mg/mL)

Vs: Sample volume (mL)

∆Abs/min: activity of the kinetic

For superoxide dismutase (SOD), a mixture of 1555 μL of Tris–DTPA buffer (50 mM, pH 8.2), 20 μL of pyrogallol (23.78 mM) diluted in HCl (10 mM), and 25 μL total cell extracts was placed in a quartz cuvette to measure the oxidation of pyrogallol at 420 nm for 1 min [17], using a Shimadzu Spectrophotometer UV-1800 (Duisburg, Germany). The activity of SOD was expressed with the following equation: % Inhibition = (∆Abs control − ∆Abs sample)/(∆Abs control) × 100

SOD activity = (% Inhibition × 2 × F)/(50 × Vs × C)

Glutathione reductase activity (GR) was quantified with the protocol developed by Staal et al. [18]: 1180 μL of 50 mM phosphate buffer^__^EDTA (6.3 mM, pH 7.4), 50 μL of total cell extracts, 35 μL of glutathione disulfide (GSSG) 5 mM, and 35 μL of nicotinamide adenine dinucleotide phosphate (NADPH; 2.4 mM), which were mixed in a quartz cuvette. Glutathione peroxidase activity (GPx) was detected by mixing 1220 μL of 50 mM phosphate buffer^__^EDTA 6.3 mM, 20 μL of total cell extracts, and 20 μL of glutathione (GSH) 10 mM. After 5 min in dark conditions, 20 μL of NADPH (2.4 mM) and 20 μL H_2_O_2_ 63.5 mM were added to the cuvette [19,20]. GR activity was read for three minutes with 60 sec of delay at 340 nm using a Shimadzu Spectrophotometer UV-1800 (Duisburg, Germany). GPx activity was measured for 3 min at 340 nm and expressed in UI/mg protein. GR activity = ((∆Abs/min) × 1.3 × F)/(0.00622 × Vs × C) GPx activity = ((∆Abs/min) × 1.3 × F)/(0.00622 × Vs × C)

0.00622 mL nmol^−1^ cm^−1^: molar extinction coefficient of NADPH 

F: dilution factor

C: Protein concentration (mg/mL)

Vs: Sample volume (mL)

∆Abs/min: activity of the kinetic

#### 2.2.6. Peroxiredoxin Expression in Neuro-2a Cells by Immunoblotting

In order to evaluate the effect of urolithin A on the expression of Prx1 and Prx3, Neuro-2a were grown in 6-well culture plates and treated with 0.5–4 μM urolithin A for 24h. Twenty-four hours later, cells were washed with PBS and scraped in lysis buffer (EDTA 1 mM, Tris 25 mM, NaCl 150 mM, 0.1% Triton; PMSF, leupeptin, and pepstatin; pH = 7.4) for 20 min. Supernatants were collected for protein determination with the bicinconinc acid method and dilutions were prepared to obtain the concentrations of proteins. Then, 10 μg of protein extract per sample was mixed with Laemmli buffer with β-mercaptoethanol, loaded onto 12% sodium dodecyl sulfate-polyacrylamide gel electrophoresis (SDS-PAGE), and subsequently transferred to nitrocellulose membranes. Immediately after this, protein transfer and loading were controlled by Ponceau red staining, followed by membrane blocking with bovine serum albumin (BSA). The following antibodies were used: anti-Peroxiredoxin 1 (ab41906; Abcam, Cambridge, UK) and anti-Peroxiredoxin 3 (AV52341; Sigma-Aldrich, St. Louis, MO, USA). Antibody binding was detected by chemiluminescence with species-specific secondary antibodies labeled with horseradish peroxidase (HRP) and visualized on a digital luminescent imager analyzer (Fujifilm LAS-4000, Cambridge, MA, USA). Images were quantified using ImageQuant TL software (Global Life Sciences Solutions, Pittsburgh, PA, USA).

### 2.3. Urolithin A and Its Role as an Inhibitor of CNS Enzymatic Targets

#### 2.3.1. Tyrosinase (TYR) Inhibition

Tyrosinase inhibitory activity of urolithin A was evaluated using 96-well plates and l-DOPA as the substrate [21]. The α-Kojic acid was used as a reference inhibitor. The reaction mixture contained 10 µL of urolithin A at different concentrations in DMSO, 80 µL phosphate buffer (pH = 6.8), 40 µL of l-DOPA, and 40 µL of tyrosinase (200 U/mL) in each well. Controls wells contained 10 µL of DMSO in place of the sample. The absorbance was measured at 475 nm (endpoint) using a Synergy H1 Hybrid Multi-Mode Reader (Biotek, Bad Friedrichshall, Germany). Inhibitory activity was determined as: % Inhibition = (Absorbance Control − Absorbance Urolitihin A)/(Absorbance Control) × 100

#### 2.3.2. Acetylcholinesterase (AChE) Inhibition

The inhibition of AChE was carried out in 96-well microplates, measuring the absorbance at 450 nm 11 times using a Synergy H1 Hybrid Multi-Mode Reader [22]. Each well contained 25 μL of ATCI (15 mM) in MilliQ water, 50 μL of buffer B (50 mM Tris-HCl, pH = 8, 0.1% bovine serum), 125 μL of DTNB (3 mM) in buffer C (50 mM Tris-HCl, pH = 8, 100 mM NaCl, 20 mM MgCl_2_), and 25 μL of urolithin A at different concentrations in buffer A (50 mM Tris-HCl, pH = 8). Finally, 25 μL of the enzyme (0.22 U/L) was added to the control and samples to complete the reaction. Blanks contained 25 μL of buffer A instead of the enzyme. Galantamine (Sigma-Aldrich, St. Louis, MO, USA), a drug used for Alzheimer’s disease, was assayed for comparative purposes as a reference inhibitor. Percentages of AChE inhibition were calculated with the following formula: % Inhibition = [1 − ((Inhibitory Slope)/(Control Slope))] × 100

#### 2.3.3. Monoamine Oxidase A (MAO-A) Inhibition

The MAO-A inhibition assay was performed following a protocol described by Olsen et al. [23]. Here, 50 µL of urolithin A at different concentrations in DMSO, 50 µL chromogenic solution (417 mM 4-aminoantipyrine, 800 µM vanillic acid, 4 U/mL horseradish peroxidase in potassium phosphate buffer pH = 7.6.), 100 µL of tyramine (300 µM), and 50 µL of MAO-A (8 U/mL) was supplemented into the well. As a standard reference inhibitor, clorgyline was selected. Control wells contained 50 µL of DMSO instead of urolithin A. The absorbance was read at 490 nm every 5 min over 30 min in a Synergy H1 Hybrid Multi-Mode Reader (Biotek, Bad Friedrichshall, Germany). Percentages of MAO-A inhibitions were calculated with the following formula: % Inhibition = [1 − ((Inhibitory Slope)/(Control Slope))] × 100

### 2.4. Urolithin A and Its Role as a Direct Free Radical Scavenger

#### 2.4.1. Oxygen Radical Antioxidant Capacity ORAC Assay 

The capacity or urolithin A to scavenge peroxyl radicals was measured by the oxygen radical antioxidant capacity (ORAC). Trolox was used as a reference standard for this assay. Therefore, data were represented as μmol Trolox equivalents (TE)/mg sample. Different concentrations of urolithin A (4.4 μM–4.4 mM) were dissolved in PBS and methanol (50:50) and placed into the wells. Urolithin A and Trolox were incubated with fluorescein (70 mM) in 96-well plates at 37 °C. Finally, AAPH (12 mM) was added and fluorescence was measured every 70 s for 1 h and 33 min at 485 nm (excitation) and 520 nm (emission), in a Synergy H1 Hybrid Multi-Mode Reader (Biotek, Bad Friedrichshall, Germany) [24].

#### 2.4.2. Superoxide Radicals Generated by Xanthine/Xanthine Oxidase (X/XO) System

Another antioxidant test was performed in order to evaluate the antioxidant activity of urolithin A using a more physiological system of superoxide radical generation [25]. Here, 22.8 µM nitroblue tetrazolium (NBT), 90 µM xanthine, and 16 mM Na_2_CO_3_ were mixed in phosphate buffer (pH = 6.9). Then, 240 µL of this cocktail was added to the well. Next, 30 µL of urolithin A and 30 µL of xanthine oxidase (168 U/L) were added to start the reaction. Before measurement, the plate was incubated for 2 min at 37 °C. The superoxide radicals’ (O_2_^−^) scavenging activity was assessed spectrophotometrically at 560 nm. The inhibitory activity of XO was also assayed spectrophotometrically at 295 nm. Gallic acid was used as a reference antioxidant compound. 

#### 2.4.3. DPPH Radical Assay

Here, 2,2-diphenyl-1-picrylhydrazyl (DPPH) is a purple free radical. Antioxidant reducing compounds are able to scavenge these free radicals, inducing a change in the color of this compound. A methanolic stock solution of DPPH (0.11 mM) was prepared and 150 µL was added together with 150 µL of different urolithin A concentrations dissolved in MeOH. Ascorbic acid and gallic acid were also measured as antioxidant standards. Control wells contained 150 µL of MeOH instead of urolithin A. The plate was incubated for 30 min under dark conditions and measured at 517 nm [26]. Radical scavenging capacity was calculated by the formula: % RSC = ((Absorbance Control−Absorbance Urolithin A))/(Absorbance Control) × 100

### 2.5. Statistical Analysis

Each experiment was performed at least three times on different days and results were expressed as the mean ± standard error (SE) of different assays. GraphPad Prism v.6 (GraphPad Software, San Diego, CA, USA) was required to perform data analyses, nonlinear regressions, and statistics (such as two-way ANOVA or one-way ANOVA). Statistical differences were detected by comparing IC_50_ values of urolithin A and reference compounds using Student’s *t*-test.

## 3. Results

### 3.1. Cytoprotective Properties of Urolithin A in Neuro-2a Cells 

#### 3.1.1. Urolithin A Improves Mitochondrial Activity in Neuro-2a Cells Subjected to Oxidative Stress (MTT Assay)

**Figure 2:**
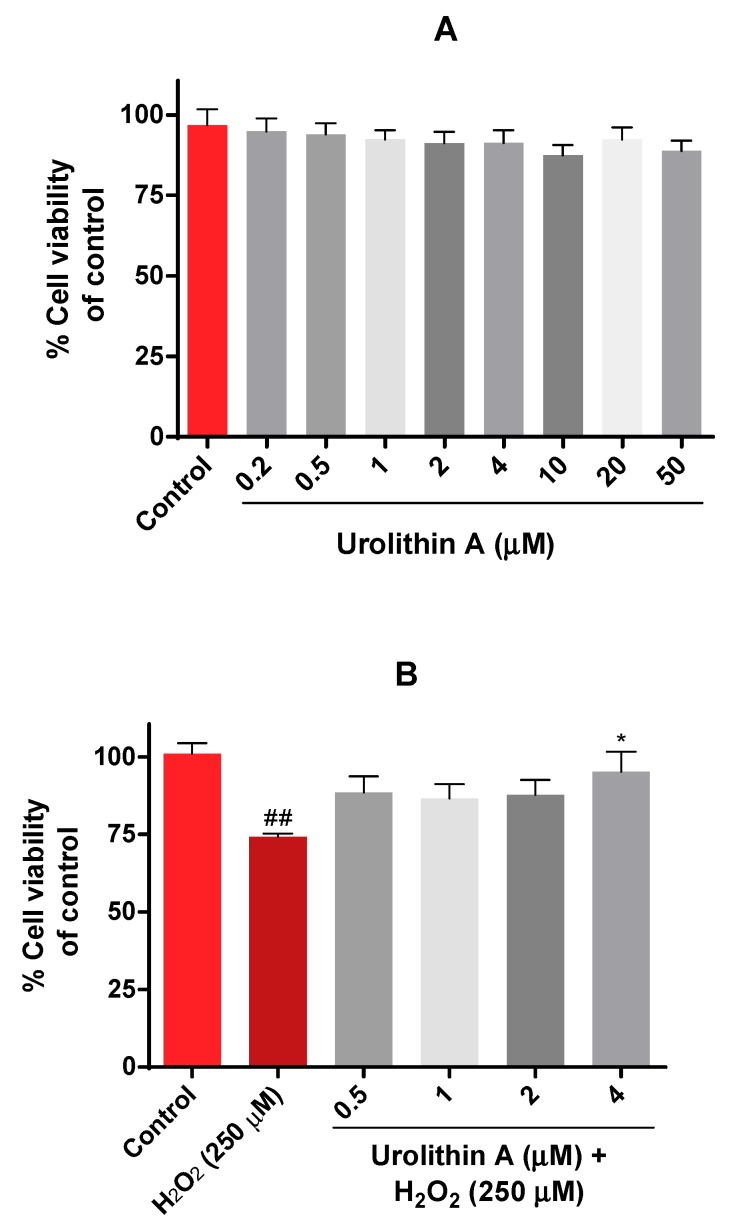
Mitochondrial activity in Neuro-2a cells culture (MTT assay). (**A**) Cytotoxicity of Neuro-2a cells after exposure to different concentrations of urolithin A. (**B**) Cytoprotective effects of urolithin A versus hydrogen peroxide (250 μM). Note: * *p* < 0.05 versus H_2_O_2_; ^##^ *p* < 0.01 versus control.

The viability of Neuro-2a cells was evaluated by the MTT assay. In this case, different physiological concentrations of urolithin (0.5–50 µM) were tested in neurons for 24 h (Figure 2A). Results were as expected because this range of concentration was non-toxic, as mitochondrial activity was not significantly reduced.

The next purpose was to evaluate the protective effects of urolithin A on Neuro-2a cells using hydrogen peroxide as a neurotoxic insult. Different conditions (100 µM to 1000 µM of H_2_O_2_) and exposure times (15, 30, 45, 60 min) determined that incubation of hydrogen peroxide for 45 min at 250 µM was the most appropriate time period for inducing oxidative stress in N2a cells. Figure 2B shows how urolithin A improves mitochondrial activity against hydrogen peroxide (250 µM) in this cell line. 

#### 3.1.2. Urolithin A Decreases Intracellular ROS Production in Neuro-2a Cells Subjected to Oxidative Stress (DCFHA-DA Assay)

**Figure 3:**
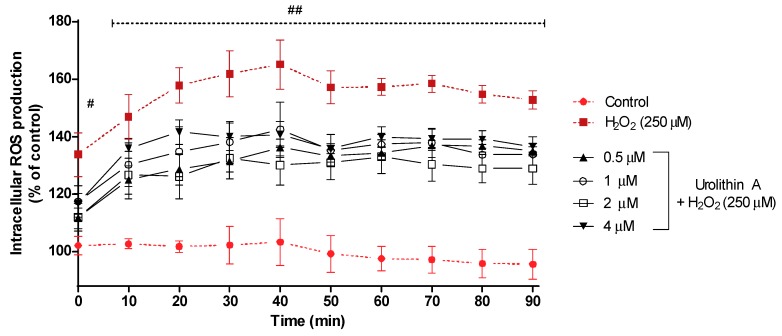
ROS production in Neuro-2a cells subjected to oxidative stress by hydrogen peroxide (250 μM) and treatments with urolithin A (0.5–4 μM). Data are expressed as percentage over control cells and the assay was carried out for 90 min in order to measure intracellular ROS production. Note: ^#^ *p* < 0.005 versus control; ^##^ *p* < 0.001 versus control. Significant differences appeared at the starting point for H_2_O_2_-N2a cells over control cells (*p* < 0.001). However, pre-treatments with 0.5 and 2 μM urolithin A at 0 and 10 min were associated with significant differences (*p* < 0.01). After 20 min, significant differences were reached at 1 μM (*p* < 0.01). Finally, 4 μM of the antioxidant exhibited significant differences (*p* < 0.01) between 40 and 60 min. Urolithin A (2 μM) displayed a greater mitochondrial response than any other co-treatment.

Figure 3 shows the intracellular ROS production for 90 min. After 40 min of exposure, intracellular ROS reached its highest formation (165%) for cells treated with hydrogen peroxide, whereas control cells (non-treated) maintained a regular level close to 100%; cells stressed with hydrogen peroxide and treated with urolithin A at different concentrations showed lower values for ROS production. Next, the transition (50 min) showed a slight decrease in ROS formation for every treatment and maintained a similar level for the rest of the experiment. 

#### 3.1.3. Urolithin A Decreases Lipid Peroxidation in Neuro-2a Cells Subjected to Oxidative Stress (Thiobarbituric Acid Reactive Species, TBARS)

**Figure 4:**
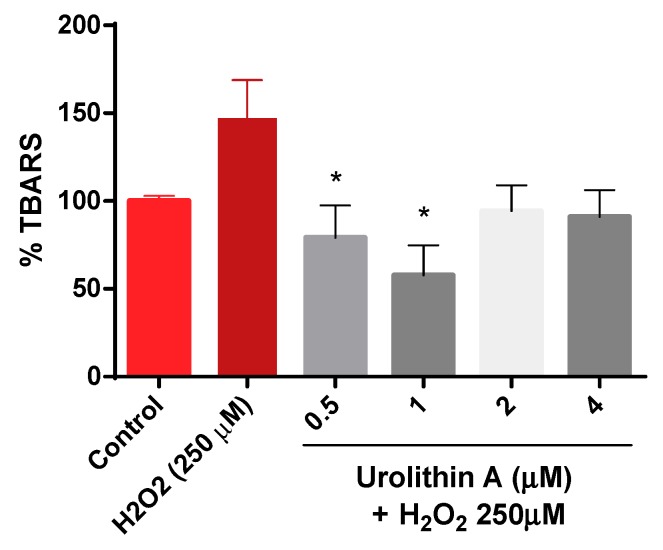
Thiobarbituric acid reactive species (TBARS) formation in Neuro-2a cells. Note: * *p* < 0.05 versus H_2_O_2_.

Figure 4 shows how thiobarbituric acid reactive species (TBARS) are generated. The results are exhibited as a percentage over the control (100%). As expected, lipid peroxidation generated by hydrogen peroxide was higher (147%) compared to control cells. Urolithin A response to lipid peroxidation was more effective at lower concentrations (0.5 and 1 µM).

#### 3.1.4. Urolithin A Enhanced the Activity of Antioxidant Enzymes in Neuro-2a Cells Subjected to Oxidative Stress (CAT, SOD, GR, GPx)

**Figure 5:**
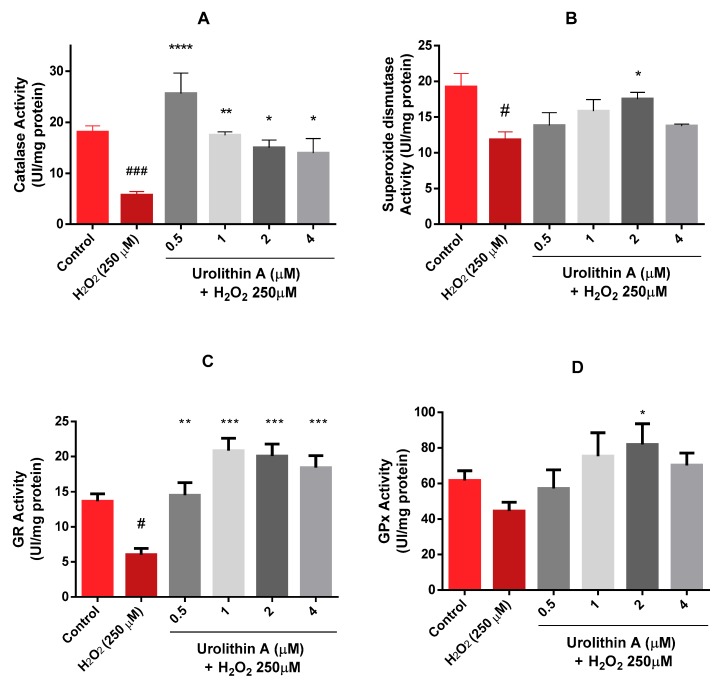
Neuro-2a cell culture redox status. (**A**) Catalase activity. (**B**) Superoxide dismutase activity. (**C**) Glutathione reductase activity. (**D**) Glutathione peroxidase activity. Note: * *p* < 0.05; ** *p* < 0.01; *** *p* < 0.005; **** *p* < 0.001 versus H_2_O_2_; # *p* < 0.05 versus Control; ### *p* < 0.005 versus Control.

Results obtained after three replications in three different lysates showed a dose-dependent tendency of catalase for the treatments with urolithin A (Figure 5A). Surprisingly, the activity of the enzyme for the lowest concentration was even higher than the control. Therefore, the in vitro antioxidant effect observed for this metabolite may be more effective at lower doses. When the cells were treated only with hydrogen peroxide, the response of catalase decreased compared to urolithin A treatments, detecting significant differences against basal cells (*p* < 0.005) and the metabolite (*p* < 0.05; 0.01; 0.001).

Different outcomes were found for the superoxide dismutase (SOD) enzyme (Figure 5B); a dose-dependent tendency appeared up to 2 µM for SOD activity. Similar activity was observed at the highest concentration as the lowest. Significant differences appeared at 2 µM (*p* < 0.05).

Comparable effects were obtained for GR and GPx activities (Figure 5C,D). Activities for 0.5 µM urolithin A looked similar to control activities for both enzymes. Each concentration of the antioxidant compound exerted significant differences for GR activity (*p* < 0.01; *p* < 0.005). However, significant differences in GPx activity were only found at 2 µM urolithin A (*p* < 0.05). 

#### 3.1.5. Peroxiredoxins Expression

**Figure 6:**
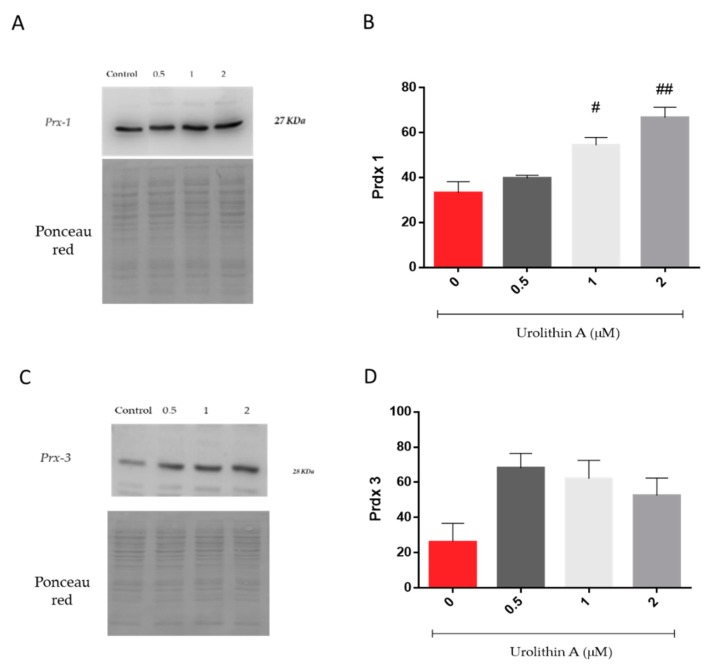
Urolithin A-induced cytoprotection of Neuro-2a cells is mediated by induction of peroxiredoxin 1 and 3 (Prx1, Prx3). Prx 1 (**A**,**B**) and Prx 3 (**C**,**D**) expressions were determined by Western blot in 10 µg of protein extract and expressed as Prx densitometry. Ponceau red staining of membranes prior to blotting was performed to further check protein transfer and loading. Note: # *p* < 0.05 versus Control; ## *p* < 0.01 versus Control

Figure 6 represents the expression of peroxiredoxins, a ubiquitous family of thiol-dependent peroxidases involved in peroxide detoxification and redox signaling [27,28]. Prx1 was increased in a dose-dependent manner over control cells (Figure 6A,B). Interestingly, mitochondrial Prx3 reached higher levels at the lower urolithin concentration tested (Figure 6C,D). Urolithin A (1 and 2 μM) treatments displayed significant differences in Prx1 expression in N2a cells over control cells (*p* < 0.05; *p* < 0.01).

### 3.2. Urolithin A Inhibits Oxidases (Monoamine Oxidase A and Tyrosinase)

As described above, the potential neuroprotective activity of urolithin A was tested on different enzymes present in the central nervous system (tyrosinase, monoamine oxidase A, and acetylcholinesterase). IC_50_ values were calculated by non-linear regression for kojic acid, clorgyline, and galantamine reference inhibitors, respectively.

**Figure 7:**
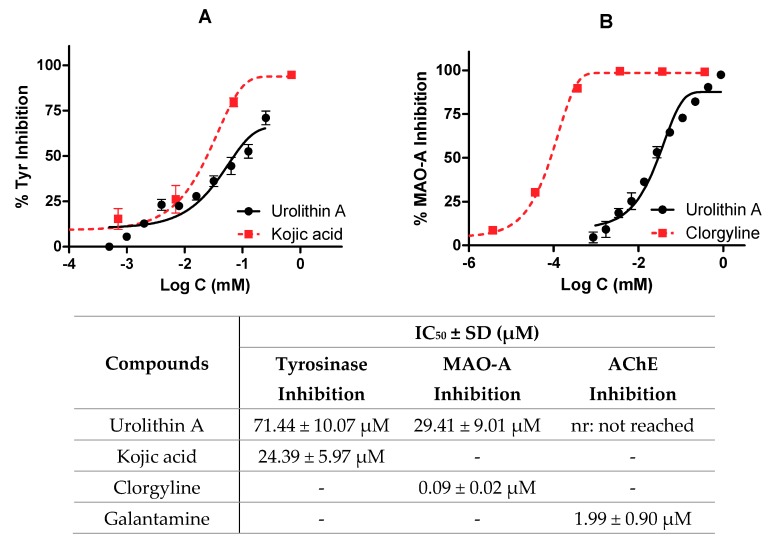
Enzymatic inhibition of urolithin A. IC_50_ values were calculated by non-linear regression. (**A**) Tyrosinase inhibition profiles of urolithin A and kojic acid. (**B**) Monoamine oxidase A (MAO-A) inhibition profiles of urolithin A and clorgyline.

Figure 7 shows the profile of urolithin A as an enzyme inhibitor; in particular, Figure 7A compares the reference inhibitor and urolithin against tyrosinase (IC_50_ values were 24.39 ± 5.97 µM and 71.44 ± 10.07 µM for kojic acid and urolithin A, respectively). Figure 7B shows how clorgyline and urolithin A are able to inhibit MAO-A. In the case of the reference inhibitor, its IC_50_ value is relatively lower than urolithin A (0.09 ± 0.02 µM and 29.41 ± 9.01 µM, respectively). Again, it is seen how the metabolite draws a dose-dependent curve.

Finally, urolithin A was not considered as an inhibitor of AChE because very high non-physiological concentrations (876 µM) were used to reach 50% of inhibition.

### 3.3. The Role of Urolithin A as a Direct Free Radical Scavenger

**Figure 8:**
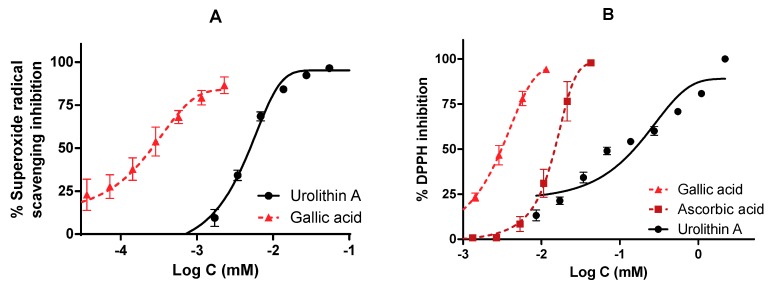

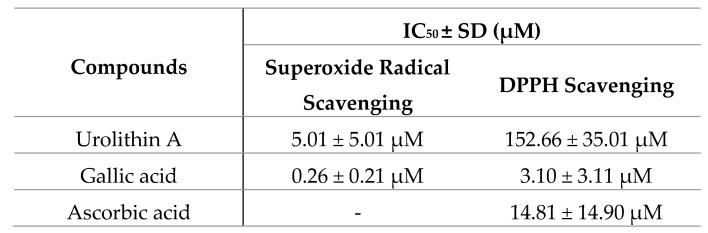
Antioxidant activity of urolithin A against physiological (superoxide) and synthetic (DPPH) radicals. IC_50_ were calculated by non-linear regression. (**A**) Urolithin A scavenges superoxide radicals generated by the xanthine/xanthine oxidase system. (**B**) DPPH inhibition of urolithin A. Gallic acid and ascorbic acid were used as reference antioxidants.

The direct antioxidant and reducing activities of urolithin A were measured by different free radical scavenging methods. To verify this fact, reference antioxidants such as gallic acid and ascorbic (vitamin C) acid were also tested and IC_50_ values were calculated (Figure 8).

The antioxidant activity measured by the ORAC assay was 13.1 µmol TE/mg for urolithin A; this result reflects a great antioxidant capacity to neutralize peroxyl radicals compared to other high-content urolithin A extracts, such as pomegranate, the activity of which was 0.49 µmol TE/mg.

On the other hand, both in the inhibition of superoxide radicals and DPPH, urolithin A showed a dose-dependent curve reaching the maximum inhibition (100%). The reference compounds achieved better activity in terms of potency. IC_50_ values were 0.26 ± 0.21 µM for gallic acid and 5.01 ± 5.01 µM for urolithin A (in the superoxide radical), while they were 3.10 ± 3.11 µM for gallic acid, 14.81 ± 14.90 µM for ascorbic acid, and 152.66 ± 35.01 µM for urolithin A (DPPH radical).

## 4. Discussion

Most of the studies involving natural products are performed with the original compounds found in the plant or food matrix, and very few are carried out with the metabolites obtained after the biotransformation in the body. Since ellagitannins are poorly absorbed in the gastrointestinal tract, urolithins have been proposed as the bioactive metabolites responsible for the beneficial effects of pomegranates and other plants containing ellagitannins. Pomegranate juice and extracts have exhibited neuroprotective properties, so here we investigate whether urolithin A, one of the major intestinal metabolites of ellagic acid, might act as a neurotherapeutic and antioxidant agent.

Neuro-2a is a neuroblastoma cell line derived from mice, which has been used because of its ability to produce microtubular proteins. Few works have been done with urolithins using this cell line, but it has been demonstrated that urolithin A protects against ischemic neuronal injury by activating autophagy [29]. This research supports our results on mitochondrial activity, demonstrating that urolithin A is not cytotoxic at this range of physiological concentrations (0.5–50 µM).

The antioxidant activity of urolithin A has already been established in different cell lines using in vitro procedures [30,31,32]. However, we here investigate whether urolithin can be considered as a neuroprotective agent due to its role as a direct free radical scavenger, as an indirect antioxidant improving the physiological antioxidant defense system of the cells, and as an inhibitor of oxidases such as monoamine oxidase A and tyrosinase.

Frequently, when the ROS and MDA levels decrease, the activity of the cytosolic enzymes (superoxide dismutase, catalase) increases; previous experiments in HepG2 cells treated with urolithin A and H_2_O_2_ have demonstrated that cells significantly increased SOD activity compared with cells subjected to oxidative stress with H_2_O_2_ [30]; in fact, SOD activity was ameliorated by urolithin in kidney cells from mice [33]. Computational studies performed with different pomegranate juice constituents revealed that they can act as pro-oxidants or antioxidants. Molecular docking studies seem to be controversial, as they have determined that urolithin A may inhibit cytosolic enzymes such as catalase, superoxide dismutase, and glutathione [34]; however, urolithin A seems to improve the activity of these enzymes in our present study. With the aim of elucidating molecular mechanisms involved in the cytoprotective and antioxidant properties of urolithin A, peroxiredoxins expression (Prx) was quantified in this work. These peroxiredoxins are antioxidant enzymes that catalyze the reduction of hydroperoxide. Prx1 and Prx3 play a great role in response to oxidative stress. In this way, the expression of Prx1 and Prx3 was increased when cells were treated with urolithin A, which translates into an antioxidant effect of urolithin A on Neuro-2a cells. Apart from the role of urolithin A as a free radical scavenger, this phenolic compound seems to act in a more specific way through the modulation of antioxidant enzymes. As observed above, urolithin A increased the expression of peroxiredoxins; this fact may explain the cytoprotective properties of urolithin A improving cell viability, decreasing ROS production, and increasing the activity of other physiological antioxidant defense systems, such as catalase, SOD, or glutathione reductase and peroxidase. Other polyphenols, such as resveratrol, have been described in the literature as antioxidant compounds with the ability to induce these cytoprotective proteins known as peroxiredoxins [35]; nevertheless, this is the first time that urolithin A is reported to increase the expression of peroxiredoxins 1 and 3.

This is also the first time that glutathione reductase and peroxidase activities were quantified in neuronal cells subjected to urolithin A treatments. In order to assess this pro-oxidant or antioxidant relationship of urolithin A, other researchers evaluated the in vitro antioxidant activity of the metabolite in HepG2 cancer cells [36]; unexpectedly, urolithin A exerted a pro-oxidant effect on these hepatoblastoma cells, however greater results were obtained in ORAC assay (6.67 ± 0.11). Regarding oxygen radical absorbance capacity, mice treated with urolithin A exhibited significantly different ORAC values after an hour of oral administration [37]. Nevertheless, urolithins may have different antioxidant potency, as shown in previous studies [38], and the ORAC value of urolithin C was lower than ours; the antioxidant properties derived from its role as a free radical scavenger were also confirmed by the DPPH in other studies [39,40,41].

ROS generation is directly linked to neurodegenerative diseases, such as Alzheimer’s, Parkinson’s, or Huntington’s diseases. In this way, after demonstrating the potential of this metabolite in Neuro2-a cells, different bioassays were carried out involving CNS enzymatic targets known as acetylcholinesterase (AChE), monoamine oxidase A (MAO-A), and tyrosinase; these enzymes are considered pharmacological targets whose inhibition may lead to neuroprotective effects. Urolithin A acted as an inhibitor of oxidase enzymes such as MAO-A and tyrosinase, thus preventing oxidative damage of certain tissues. A human study in older adults with mild memory complaints suggests that 8 ounces of pomegranate juice taken daily over one-month improves verbal memory and alters neural activity during a visual source memory task [42]. Synthetized urolithins have also demonstrated comparable activity to AChE inhibitors such as rivastigmine, galantamine, and donepezil [43]. In silico computational studies predicted that urolithins can penetrate the blood-brain barrier, preventing β-amyloid fibrillation in a *C. elegans* model [44]. In addition, urolithin A has been identified as a proper anti-inflammatory and anti-ageing compound [38]. Recently, 10 μM urolithin A was demonstrated to possess depigmentation efficacy by suppressing tyrosinase activity, attenuating melanogenesis in B16 melanoma cells [45]. Tyrosinase has been recognised as a potential pharmacological target because an excess of tyrosinase activity or dopamine may lead to neurotoxicity through dopamine quinone formation; in this sense, tyrosinase inhibitors might have protective properties in neuronal cells. MAO-A is responsible for the catalytic oxidative deamination of monoamines generating hydrogen peroxide; for this reason, MAO-A inhibitors have been proposed as neuroprotective agents as well. The inhibitory activity of pomegranate polyphenols on MAO-A has already been tested, which could explain its effects in the CNS [22]; however, to the best of our knowledge this is the first report of urolithin A as an antioxidant agent capable of neutralizing MAO-A oxidative reactions.

## 5. Conclusions

Urolithin A, a gastrointestinal metabolite from ellagitannins, is a promising therapeutic antioxidant agent with potential pharmaceutical or food applications for the prevention of oxidative stress-associated disorders, probably though the improvement of the cell antioxidant capacity by increasing the expression of thiol-dependent peroxidases.
